# Phosphate limitation induces the intergeneric inhibition of *Pseudomonas aeruginosa* by *Serratia marcescens* isolated from paper machines

**DOI:** 10.1111/1574-6941.12086

**Published:** 2013-03-11

**Authors:** Pei-An Kuo, Chih-Horng Kuo, Yiu-Kay Lai, Peter L Graumann, Jenn Tu

**Affiliations:** 1 Institute of Microbiology, University of FreiburgFreiburg, Germany; 2 Institute of Biotechnology, College of Life Science, National Tsing Hua UniversityHsinchu, Taiwan; 3 Institute of Plant and Microbial Biology, Academia SinicaTaipei, Taiwan; 4 SYNMIKRO, University of MarburgMarburg, Germany

**Keywords:** phosphate limitation, intergeneric inhibition, bacterial competition, bacteriocin, biofilm development, *Serratia marcescens*

## Abstract

Phosphate is an essential nutrient for heterotrophic bacteria, affecting bacterioplankton in aquatic ecosystems and bacteria in biofilms. However, the influence of phosphate limitation on bacterial competition and biofilm development in multispecies populations has received limited attention in existing studies. To address this issue, we isolated 13 adhesive bacteria from paper machine aggregates. Intergeneric inhibition of *Pseudomonas aeruginosa* WW5 by *Serratia marcescens* WW4 was identified under phosphate-limited conditions, but not in Luria–Bertani medium or M9 minimal medium. The viable numbers of the pure *S. marcescens* WW4 culture decreased over 3 days in the phosphate-limited medium; however, the mortality of *S. marcescens* WW4 was significantly reduced when it was co-cultured with *P. aeruginosa* WW5, which appeared to sustain the *S. marcescens* WW4 biofilm. In contrast, viable *P. aeruginosa* WW5 cells immediately declined in the phosphate-limited co-culture. To identify the genetic/inhibitory element(s) involved in this process, we inserted a mini-Tn5 mutant of *S. marcescens* WW4 that lacked inhibitory effect. The results showed that an endonuclease bacteriocin was involved in this intergeneric inhibition by *S. marcescens* WW4 under phosphate limitation. In conclusion, this study highlights the importance of nutrient limitation in bacterial interactions and provides a strong candidate gene for future functional characterisation.

## Introduction

In natural environments, the growth of heterotrophic bacteria is often limited by nutrients, such as organic carbon, inorganic nitrogen and inorganic phosphorus. For instance, inorganic phosphorus has been frequently reported to restrict the biomass and production of heterotrophic bacterioplankton in different aquatic ecosystems (Farjalla *et al*., 2002; Granéli *et al*., 2004; Jansson *et al*., 2006) or to regulate the distribution of bacterial subgroups with different nucleic acid contents in a freshwater hypolimnion (Nishimura *et al*., 2005). Phosphate limitation also affects the physiology of bacteria growing in biofilms, for example changing the structure of *Serratia marcescens* MG1 biofilm (Rice *et al*., 2005). In addition, it up-regulates the expression of the Pho regulon, which results in *Pseudomonas aureofaciens* PA147-2 (Monds *et al*., 2001) and *Vibrio cholerae* (Sultan *et al*., 2010), losing the ability to form biofilm. Therefore, phosphate limitation influences different types of physiological responses and population dynamics of bacteria in biofilms and bacterioplankton in pelagic ecosystems.

Phosphate limitation represents a particular problem for bacteria that live in biofilms. The proximity of bacteria with neighbouring cells usually enhances competition for limited space and nutrients (such as phosphate). To be successful in this highly competitive environment, one effective strategy is to inhibit competitors by producing antimicrobial molecules or a cocktail of deleterious compounds. Such compounds include biosurfactants, toxic chemicals, secondary metabolites, lytic enzymes, antibiotics or bacteriocins (Shank & Kolter, 2009; Hibbing *et al*., 2010). Most of these antibacterial molecules, such as antibiotics, often function over a broad spectrum of targets to directly antagonise competitors and sometimes even act against eukaryotes. In contrast, the three known types of bacteriocins (i.e. RNA inhibitors, endonucleases or pore-forming toxins) target specific competitors (Riley & Wertz, 2002). Due to the immunity protein in host cells, ribosomal synthesised bacteriocins only inhibit the growth of closely related target bacteria. Hence, in the bacterial community, bacteriocins enhance the competitive ability of bacteria to survive in challenging surroundings, in addition to blocking the invasion of neighbouring competitors to facilitate a stable coexistence. One known example is *Escherichia coli* bacterial populations carrying a DNA-degrading bacteriocin (colicin E2 or E7; Majeed *et al*., 2011).

Previous studies of bacterial competition and biofilm formation primarily investigated interspecific bacteria under laboratory conditions. However, in a natural ecosystem, different species and genera of heterotrophic bacteria often coexist, facing similar nutrient limitation (especially with respect to phosphate; Granéli *et al*., 2004). Such bacteria may exhibit highly variable behaviours and physiological responses to overcome phosphate limitation and intergeneric competition. Yet, the actual mechanism of the production of antimicrobials for competitive purposes under phosphate limitation is often overlooked by researchers. Furthermore, the effect of phosphate limitation on the biofilm development of intergeneric bacteria is rarely discussed. The papermaking industry is often troubled with biofilm problems, which cause huge financial losses. For example, biofilms, which are commonly called ‘slime-producing bacteria’, in paper machines attach to papermaking materials or machine parts and may clog wires and felts, causing the continuously forming paper sheet to break, and interrupting production. In another example, biological infections in the form of biofilm bacteria are extremely difficult to eradicate due to the high resistance to antibiotics.

In this study, we isolated biofilm-forming bacteria from biofilm slurry in the spray water system of paper machines. Paper mill process waters are low in nutrients and form a phosphate-limited environment (e.g. the phosphate content can be as low as 1.1 mg L^−1^; Vaisanen *et al*., 1998). Although this environment is artificial, bacterial interactions among community members are representative of a natural aquatic-biofilm ecosystem. To explore bacterial competition and biofilm formation in paper machines, we constructed a microcosm of two bacterial species and tested bacterial interactions and biofilm development under different levels of phosphate limitation. The results of this study are considered in relation to bacterial intergeneric competition in natural environments and contribute knowledge to control biofilm problems caused by bacteria in the papermaking industry.

## Materials and methods

### Isolation of bacterial strains from paper machines

Three samples of turbid spray water containing pulp, cellulose fibres and bacterial aggregates were scraped and collected from the wire spray system of three paper machines at the Yuen-Foong-Yu Paper Manufacturing Company in Taiwan (one sample per sampling machine). The sticky aggregates in the samples were collected by centrifuging the samples for 10 min at 200 ***g*** and were then dispersed in 50 mL of sterile saline. Fibres in the suspension were removed by centrifuging the suspension for 10 min at 200 ***g***. To isolate bacterial cells with adhesive properties, sterile stainless steel slides were immersed in the resulting supernatant. After 4-h incubation at 37 °C (which represents an approximate temperature to that in the water tank of wire spray system; Vaisanen *et al*., 1998), the slides were carefully scraped with sterile cotton swabs. The adhesive bacteria were washed with sterile saline, spread on Luria–Bertani (LB) plates and left to grow at 37 °C overnight. Individual isolates were repeatedly streaked until only one colony type was visible. Then, only the isolates that had same 16S rRNA gene sequences in all three sample replicates were selected as isolated strains.

### 16S ribosomal RNA gene sequence analysis of bacterial isolates

The partial 16S rRNA gene fragment of each isolate was amplified using the primer pair 5′-AGAGTTTGATCCTGGCTCAG-3′ and 5′-AAGGAGGTGATC(C/A)(A/G)CCGCA-3′ [between nucleotide positions 8 and 1527 (*E. coli* K-12 16S rRNA gene sequence numbering)] according to Weisburg's procedure (Weisburg *et al*., 1991). The PCR products were purified using QIAquick PCR purification kit (#28106; Qiagen, Germany). All DNA samples were sequenced using BigDye Terminator v1.1, and DNA sequencing reactions were resolved using an ABI 3730xl DNA Analyzer. Sequence analysis was performed using the software Vector-NTI and the NCBI database blast (http://blast.ncbi.nlm.nih.gov). The most significant hit found in the NCBI GenBank was used to infer the taxonomy of each newly isolated strain (Table 1). All 16S rRNA gene sequences from this study were deposited in the GenBank database, and the accession numbers are listed in Table 1.

**Table 1:** Identification of adhesive bacterial isolates from the biofilm slurry in paper machines

Isolates	GenBank accession number	Best blast hit	% identity
WW1	EF433544	*Comamonas* sp. JS-6	99.9
WW2	EF433545	*Klebsiella* sp. R-21934	99.5
WW3	EF433546	*Corynebacterium vitarumen*	97.6
WW4	EF491732	*Serratia marcescens*	99.9
WW5	EF433547	*Pseudomonas aeruginosa*	99.9
WW6	EF433548	*Pseudomonas* sp. LB-2	99.8
WW7	EF433549	*Aeromonas* sp. NLEP A-1607	99.7
WW8	EF433550	*Bacillus pumilus*	99.9
WW9	EF433551	*Pseudomonas* sp. J11	99.5
WW11	EF433552	*Sphingomonas* sp. MN	96.9
WW12	EF433553	*Exiguobacterium* sp. BTAH1	100.0
WW16	EF433554	*Enterobacter* sp. WAB1938	99.6
WW21	EF433555	*Acinetobacter* sp. DR.Y12	99.9

### Bacterial culture media

The broth culture media included LB nutrient medium (Difco #244610; BM company), M9 minimal salts medium [M9; 18.7 mM NH_4_Cl, 8.5 mM NaCl, 47.7 mM Na_2_HPO_4_, 22 mM KH_2_PO_4_, supplemented with 0.5 mM MgCl_2_·6H_2_O, 0.33 mM CaCl_2_·2H_2_O, 0.2% (w/v) glucose and 0.01% (w/v) yeast extract] (Maniatis *et al*., 1989) and BM enriched basal medium [BM; 18.7 mM NH_4_Cl, 1.7 mM NaCl, 2.3 mM K_2_HPO_4_, 0.5 mM MgCl_2_·6H_2_O, 0.33 mM CaCl_2_·2H_2_O, modified with 0.2% (w/v) glucose and 0.01% (w/v) yeast extract] (Vaisanen *et al*., 1998; Huang *et al*., 2009).

To confirm the effect of phosphate limitation on bacterial inhibition, the phosphate level of M9 phosphate-reduced (M9 P-reduced) medium was reduced to the same level as that in BM medium (2.3 mM). BM medium was supplied with half (BM+1/2P; 36 mM) or an equivalent (BM+P; 69.7 mM) amount of phosphate as that in M9 medium.

### Bacterial viability in pure or co-cultures

The preculture of *S. marcescens* WW4 or *Pseudomonas aeruginosa* WW5 was grown overnight at 37 °C in each testing broth medium detailed in the previous section. Bacteria were centrifuged for 15 min at 3500 ***g***, and resuspended in fresh medium at a turbidity of OD_600_ 0.1 for pure culture experiments. For co-culture experiments, *S. marcescens* WW4 and *P. aeruginosa* WW5 were mixed at a final turbidity of OD_600_ 0.1, which had the approximate cell concentrations of *c*. 10^8^ CFU mL^−1^ of *S. marcescens* WW4 and *c*. 9.5 × 10^7^ CFU mL^−1^ of *P. aeruginosa* WW5. The pure or co-cultures were incubated at 37 °C and 90 r.p.m. The viable red colony number (CFU mL^−1^) of *S. marcescens* WW4 was counted on LB plates at 0, 3, 6, 12, 24, 48 and 72 h. The nalidixic acid (Nx)-containing LB plates (20 mg L^−1^) were used to count the viability of Nx-resistant *P. aeruginosa* WW5. All viability data were counted for each dilution, with three replicated plates at each time point. The live/dead status of bacteria was examined using propidium iodide nucleic acid stain (#P-3566; Molecular Probes) and observed under a fluorescence microscope (Axio Imager Z1; Zeiss). The excitation/emission maxima for propidium iodide were about 488 nm/617 nm. For the concentrated *S. marcescens* WW4 viability experiment, *S. marcescens* WW4 cells were inoculated at different cell concentrations, specifically 0.1, 0.2, 0.3, 0.4, 0.5 or 0.6 of OD_600_. The concentrated *S. marcescens* WW4 were co-cultured with or without *P. aeruginosa* WW5 (OD_600_ 0.1) in BM medium, and the viability of *S. marcescens* WW4 was counted after 24 h. Means and standard errors of all data were calculated from five experimental replicates. Significance was calculated using a two-tail *t*-test.

### Biofilm-forming experiment in chambers

The experiment to form biofilm in chambers was modified from the flow cell design described by Wolfaardt *et al*. (1994). The flow cell consisted of an acrylic chamber (45 × 65 × 15 mm in size), which had an internal dimension of 37 × 55 × 9 mm and a working volume of *c*. 18.8 mL. An 18 × 35 × 3.5 mm window at the top of the chamber was mounted with a microscope glass cover slip (32 × 50 mm, Thickness No. 1). The chambers were sterilised overnight with 70% ethanol before use.

*Serratia marcescens* WW4 grown in BM medium at 37 °C overnight was diluted to OD_600_ 0.1 with fresh medium and poured into a sterile chamber for pure culture experiments. For co-culture experiments, *P. aeruginosa* WW5 (OD_600_ 0.1) was mixed with *S. marcescens* WW4 (final turbidity of OD_600_ 0.1 or 0.01) in BM medium in the sterile chamber. Tape was used to seal a sterile glass cover slip over the hole of the chamber. The biofilm-forming sets were agitated on a three-dimensional rocking shaker and incubated at 37 °C. Attached cells of *S.marcescens* WW4 in chambers were stained with 4′,6-diamidino-2-phenylindole (DAPI; 1 μg mL^−1^) and observed on the first, third and sixth day, using a fluorescence microscope (Axio Imager A1; Zeiss) at 400 × magnification, and with the standard DAPI filter set. Twenty photographs per time point were taken at random locations on the cover slips of four different chambers (five photographs in each chamber, covering about 1 mm^2^). All image data were analysed using ImageJ software (National Institutes of Health). The image thresholds were manually adjusted to the same status of proper area coverage, and the fraction (%) of the adhesive area where *S. marcescens* WW4 was located was calculated using the ImageJ program. All adhesive fraction data were analysed in triplicate to minimise operational error.

### Random insertion mutagenesis of *S. marcescens* WW4 with mini-Tn5 transposon

*Serratia marcescens* WW4 was subjected to random transposon mutagenesis using a mini-Tn5 transposon, constructed on the pUT suicide vector as described by De Lorenzo *et al*. (1990). The pUT plasmid carrying mini-Tn5 lacZ1 was maintained in an *E. coli* S17 (λ pir) donor strain and introduced into *S. marcescens* WW4 by conjugal transfer, according to the spot mating method (Winson *et al*., 1998). In brief, tetracycline-resistant *S. marcescens* WW4 and *E. coli* S17 (λ pir) were mixed at a ratio of 1 : 10 in LB medium at 37 °C for 6 h. Transconjugates were isolated on BM plates containing kanamycin (75 mg L^−1^) for the transposon-inserted mutant selection, and tetracycline (20 mg L^−1^) was added to counter-select against the *E. coli* donor. The insertion mutants that had lost inhibitory ability against *P. aeruginosa* WW5 were screened in 96-well plates by incubating *S. marcescens* transconjugates with *P. aeruginosa* WW5 in BM medium at 37 °C for 24 h, and the viability of *P. aeruginosa* WW5 was then counted. The mutants that were identical to the *P. aeruginosa* WW5 viability approximating the pure culture control were selected as candidates. The candidate mutants were assayed three times.

### Identification of mutated gene in the genome of the *S. marcescens* WW4 mutant

The identification of the transposon-mediated insertional locus in the genome of *S. marcescens* WW4 mutants was carried out as described by Martin & Mohn (1999). To sequence the genomic DNA sequences flanking the mini-Tn5 insertion, the genomic DNA of mutant was digested with *Bam*HI followed by ligation. The flanking regions around the transposon were amplified by inverse PCR with a pair of PCR primers, which were located inside the two terminal repeats of mini-Tn5, and designed to extend outward. Amplified genomic flanking fragments were sequenced.

To determine the genomic context of transposon insertion in the mutant, the sequence from the flanking region was used as query to perform a blastn search (Altschul *et al*., 1990; Camacho *et al*., 2009) against the complete *S. marcescens* WW4 genome sequence (GenBank accession number CP003959).

## Results and discussion

### Isolation of adhesive bacteria from the biofilm of paper machines

To investigate species interactions and biofilm formation in a phosphate-depleted ecosystem, we isolated 13 adhesive bacteria from the slimy water in the wire spray system of paper machines. The strains were identified by comparing their partial 16S rRNA gene sequence to available references in the NCBI GenBank (Weisburg *et al*., 1991). Based on the blastn results, each of the 13 isolates was classified to the genus or species level (Table 1). After examining the ability of these strains to be cultured and form biofilm, only *S. marcescens* WW4, *P. aeruginosa* WW5, *Pseudomonas* sp. WW6 and *Aeromonas* sp. WW7 formed sufficient aggregation on the cover slips in the phosphate-limited BM medium, which was similar to the spray water conditions, comprising low nutrient and phosphate levels (Vaisanen *et al*., 1998). Of these four bacteria, only *S. marcescens* WW4 and *P. aeruginosa* WW5 formed mature biofilm structures. Therefore, *S. marcescens* WW4 and *P. aeruginosa* WW5 were selected as models for describing one of many possible intergeneric interactions that might occur in phosphate-limited co-cultures. These two bacteria were selected because they demonstrated the best biofilm-forming ability of the 13 isolates.

Out of the 13 isolates, bacteria similar to the isolates *Comamonas* sp.WW1, *S. marcescens* WW4, *P. aeruginosa* WW5, *Bacillus pumilus* WW8 and *Enterobacter* sp.WW16 have been described to have biofilm-forming ability in varied environments (Vaisanen *et al*., 1998; Kolari *et al*., 2001; Andersson *et al*., 2008; Wu *et al*., 2010). Furthermore, relatives of the isolates *Pseudomonas* sp. WW6 and *Acinetobacter* sp.WW21 have been shown to co-aggregate with other bacteria to form mixed biofilms (Wolfaardt *et al*., 1994; Simoes *et al*., 2008). Therefore, many isolates might a have high potential for forming biofilm in paper machines. For instance, *S. marcescens* WW4, *P. aeruginosa* WW5, *Pseudomonas* sp. WW6 and *Aeromonas* sp.WW7 form thick aggregations in BM medium, indicating that these strains are adapted to grow and form biofilm in a phosphate and nutrient-limited environment. *Serratia marcescens* WW4 and *P. aeruginosa* WW5 are particularly suited to such environments.

### Inhibitory effect of *P. aeruginosa* WW5 by *S. marcescens* WW4 in phosphate-limited growth medium

**Fig. 1:**
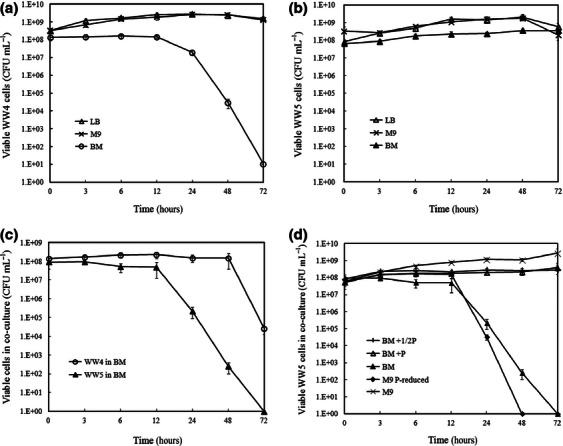
The viability of *Serratia marcescens* WW4 and *Pseudomonas aeruginosa* WW5 in pure or co-cultures. The viability of *S. marcescens* WW4 (a) or *P. aeruginosa* WW5 (b) pure culture in LB, M9 or BM medium was counted at 0, 3, 6, 12, 24, 48 and 72 h. The individual viability of co-cultured *S. marcescens* WW4 and *P. aeruginosa* WW5 in BM medium is shown in (c). To confirm the effect of phosphate, the viability of *P. aeruginosa* WW5 in co-culture is shown in (d), when cultured in phosphate-supplied BM medium (BM+1/2P or BM+P) or phosphate-reduced M9 medium (M9 P-reduced), compared with BM or M9 medium. All data show the means of viable cell numbers (CFU mL^−1^), and the bars represent the standard error.

To approximate the nature of bacterial intergeneric interactions in industrially processed water, we cultivated *S. marcescens* WW4 and *P. aeruginosa* WW5 in phosphate-limited BM medium and recorded variation in individual viabilities of the two bacteria over a 3-day period. Due to *S. marcescens* WW4 having a red pigmentation and *P. aeruginosa* WW5 being Nx-resistant, these two bacteria could be easily distinguished by visual colony counts and antibiotic selection. We also compared the growth of two bacteria in LB and M9 medium with BM medium. The viability curves showed that *S. marcescens* WW4 grew well in LB and M9 medium over a 3-day period, but not in BM medium. The viable cell number of *S. marcescens* WW4 in BM medium significantly decreased after 24 h and finally declined to 11 CFU mL^−1^ on the third day (Fig. 1a). In contrast, *P. aeruginosa* WW5 grew well in all three media until the third day of incubation (Fig. 1b). However, in the co-culture of *S. marcescens* WW4 and *P. aeruginosa* WW5 in BM medium, the viability of *S. marcescens* WW4 was maintained at about 10^8^ CFU mL^−1^ for 2 days. On the third day, the viable number of *S. marcescens* WW4 declined to 4 × 10^4^ CFU mL^−1^. In contrast, the viability of *P. aeruginosa* WW5 in the BM medium co-culture dramatically declined after 12 h. On the third day, the viable number of *P. aeruginosa* WW5 was no longer detectable (< 1 CFU mL^−1^; Fig. 1c). Meanwhile, in the LB and M9 medium co-cultures, both *S. marcescens* WW4 and *P. aeruginosa* WW5 grew well and remained viable throughout the 3 days of incubation. The results indicate that *S. marcescens* WW4 exhibited an intergeneric inhibition on *P. aeruginosa* WW5 when two bacteria were co-cultured in phosphate-limited BM medium, and that the viability of the *S. marcescens* WW4 population was maintained under phosphate limitation when *P. aeruginosa* WW5 was present.

To examine the effect of phosphate in culture media, we increased the phosphate content in BM medium to half (BM+1/2P medium) or the equivalent (BM+P medium) concentration as that in M9 medium. The viability of *P. aeruginosa* WW5 in the co-cultures of the phosphate-supplied BM (BM+1/2P and BM+P) media was kept under the same conditions as the pure cultures for 3 days. Interestingly, *P. aeruginosa* WW5 grew well for 3 days in the M9 medium co-culture. However, when the phosphate concentration of the M9 medium was reduced, the viability of *P. aeruginosa* WW5 in the co-culture significantly decreased in the phosphate-reduced M9 medium (M9 P-reduced) after the first day (Fig. 1d). This phenomenon was similar to that observed in the BM medium co-culture. Based on the data, we concluded that phosphate is a critical factor for the intergeneric inhibition in the co-cultures of *S. marcescens* WW4 and *P. aeruginosa* WW5.

In an industrial bacterial ecosystem, phosphate limitation influences the growth of bacteria (Vadstein *et al*., 2003) and the manner of bacterial competition, which is supported by this study. We demonstrated the coexistence of *S. marcescens* WW4 and *P. aeruginosa* WW5 under rich-nutrient conditions (with no antagonistic antibiotic responses being recorded); however, the bacteria exhibited another interaction under phosphate limitation. The strong opportunistic pathogen *P. aeruginosa* has often been reported to efficiently compete with many genera of bacteria, including *Klebsiella pneumoniae*, *Burkholderia cepacia*, *Hyphomicrobium* sp. and *Agrobacterium tumefaciens* (Banks & Bryers, 1991; Stewart *et al*., 1997; Al-Bakri *et al*., 2004; An *et al*., 2006). The growth of *P. aeruginosa* WW5 in pure culture remained strong in BM medium, compared with *S. marcescens* WW4 (Fig. 1a and b). However, intergeneric inhibition only occurred in co-cultures with phosphate and nutrient-limited conditions (similar to the conditions in paper machines), rather than in rich medium (LB) and in normal minimal salts medium (M9). Hence, under phosphate limitation, *S. marcescens* WW4 dramatically overcame the vigorous *P. aeruginosa* WW5.

### Biofilm development of *S. marcescens* WW4 in co-cultures

To simulate biofilm formation in the spray water reservoir of paper machines and to continuously observe the development of the biofilm structure, we designed a biofilm-forming chamber adapted from the study by Wolfaardt *et al*. (1994). The cells were agitated during incubation to allow the media and a little air to mix. Bacteria could adhere to the glass cover slip at the top of the chamber, which served as the liquid–air interface; therefore, this surface provided the best growth conditions within the chamber.

**Fig. 2:**
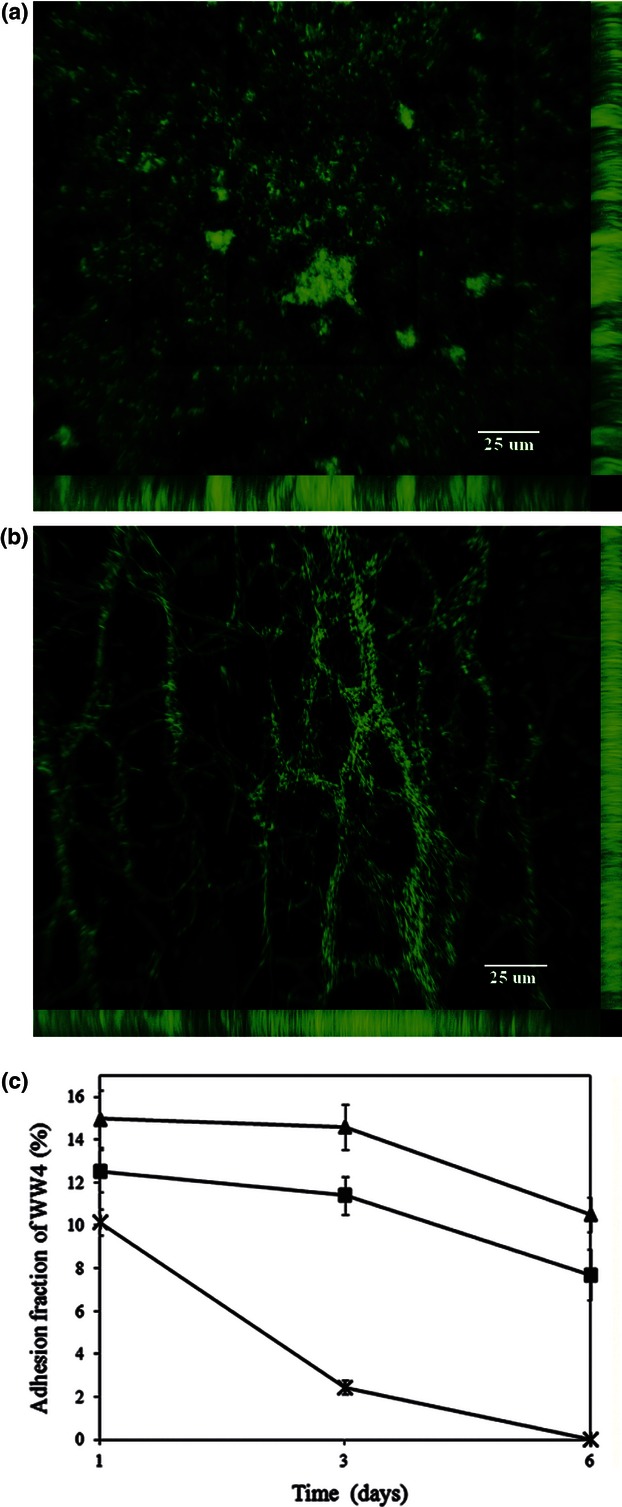
The biofilm of *Serratia marcescens* WW4 and *P. aeruginosa* WW5 in chambers, and the adhesion fraction of *S. marcescens* WW4 in pure or co-cultures. The hill-like biofilm structure of *P. aeruginosa* WW5 (a) and the filamentous biofilm structure of *S. marcescens* WW4 (b) were observed in BM medium in the biofilm-forming chambers. The pictures were obtained from 1-day DAPI-stained cultures, and the bars represent 25 μm. (c) The biofilm of *S. marcescens* WW4 mixing in the absence (**×**) or presence of equal (▲) or tenfold (▪) *P. aeruginosa* WW5 was incubated in phosphate-limited chambers. The adhesion fraction of *S. marcescens* WW4 was photographed at 1, 3 and 6 days using a fluorescence microscope, and the images were analysed using ImageJ software. All data show the means and standard errors of the adhesion proportion (%) of *S. marcescens* WW4 biofilm.

Both *S. marcescens* WW4 and *P. aeruginosa* WW5 successfully adhered to the cover slips, forming integrated biofilm structures, even under phosphate limitation. In the phosphate-limited biofilm-forming chamber, *P. aeruginosa* WW5 formed a hill or tower-like biofilm structure (Fig. 2a), while *S. marcescens* WW4 formed an intricate three-dimensional biofilm architecture containing long fibres and cell chains, which were sometimes web-like (Fig. 2b). Interestingly, the two types of biofilm were noticeably different and highly distinguishable. When *S. marcescens* WW4 was solely cultured in BM medium, the biofilm was fully constructed in the chamber on the first day, forming a structure similar to the filamentous biofilm described in reports about the interaction between *S. marcescens* and pathogenic protozoa or parasites (Castro *et al*., 2007; Moraes *et al*., 2008). We found that the adhesion fraction of *S. marcescens* WW4 was similar to its viability under phosphate limitation. The proportion of *S. marcescens* WW4 that was aggregated on the cover slips declined from 10% to 2% in 3 days, with no aggregate being detected on the sixth day (Fig. 2c). The phosphate-dependent phenomenon of *S. marcescens* WW4 is probably due to quorum sensing (i.e. cell density control) and nutrient levels being involved in the detachment of its filamentous biofilm, as described by Rice *et al*. (2005) for *S. marcescens* MG1 after 75 h.

However, when *S. marcescens* WW4 was co-cultured with *P. aeruginosa* WW5 at a ratio of 1 : 1 or 1 : 10 in BM medium chamber, the adhesion fractions of *S. marcescens* WW4 were maintained at 8–10% after 6 days (Fig. 2c). In addition, the pure biofilm of *P. aeruginosa* WW5 was preserved well in BM medium for more than 6 days. However, in the co-cultured BM medium chambers, hill-like aggregates of *P. aeruginosa* WW5 were not detected during the 6-day period (only *S. marcescens* WW4 filaments were available for quantification). Hence, photographs containing the two biofilms were not available from the phosphate-limited co-culture chamber. The viability of *S. marcescens* WW4 and *P. aeruginosa* WW5 in the co-culture chamber was also monitored and showed the same variation as the adhesion fractions of both bacteria (Supporting Information, Fig. S1). This phenomenon might indicate that lowered levels of adhesions are associated with the quantity of viable cells. Thus, phosphate limitation also acted on the biofilm development of *S. marcescens* WW4, which caused intergeneric inhibition by suppressing the surface attachment of *P. aeruginosa* WW5 in the co-culture chambers. Furthermore, the presence of *P. aeruginosa* WW5 supported the biofilm architecture of *S. marcescens* WW4 for longer under phosphate limitation.

In our phosphate-limited chambers, the effect of intergeneric inhibition was apparent in the biofilm formation and development of both bacteria. Most importantly, our chamber system showed that an intricate interplay existed between the two different bacterial genera on the surface under phosphate limitation. It is clear that competition for surface area is intensive. Even when a single species wins to initiate biofilm formation, it may be quickly outcompeted by a different species. For example, in the reconstitution of biofilms in a river, bacteria and filamentous cyanobacteria were first to pioneer the surface, but were later outcompeted by diatoms and green algae (Besemer *et al*., 2007). Therefore, it is speculated that disadvantaged *S. marcescens* WW4 probably cannot maintain population size and biofilm structure for long under phosphate-limited conditions. Hence, to compete under phosphate limitation, *S. marcescens* WW4 might kill *P. aeruginosa* WW5 or obtain certain growth factors to support its viability to occupy this biofilm habitat.

### Reduced death of concentrated *S. marcescens* WW4 by *P. aeruginosa* WW5

When *S. marcescens* WW4 was individually cultured in BM medium, the turbidity was often below OD_600_ 0.35, and the viable cells of pure *S. marcescens* WW4 culture rapidly decreased after 24 h. However, when *S. marcescens* WW4 was co-cultured with *P. aeruginosa* WW5, its viability did not decrease until 48 h had passed (Fig. 1a and c). To confirm the influence of *P. aeruginosa* WW5 on *S. marcescens* WW4 under phosphate limitation, we used different initial concentrations of *S. marcescens* WW4 cells (0.1, 0.2, 0.3, 0.4, 0.5 and 0.6 of OD_600_) and co-incubated the concentrated *S. marcescens* WW4 in the absence and presence of *P. aeruginosa* WW5 (OD_600_ 0.1) in BM medium. After 24 h, the viability of the pure concentrated *S. marcescens* WW4 dramatically decreased to < 0.08% in experiments of initial OD_600_ 0.3, 0.4, 0.5 and 0.6 (Table 2). However, when *S. marcescens* WW4 was co-cultured with *P. aeruginosa* WW5, there was a significant increase in the percentage of viable concentrated *S. marcescens* WW4 (Table 2), especially in the initial conditions of OD_600_ 0.3. The significance between the pure cultures and co-cultures was *P* < 0.001 for initial OD_600_ 0.3 and *P* < 0.05 for initial OD_600_ 0.4, 0.5 and 0.6. Furthermore, we also examined the survival status (i.e. live/dead cells) of concentrated *S. marcescens* WW4 and *P. aeruginosa* WW5 in co-cultures using propidium iodide nucleic acid stain after 24 h. Microscopic observations showed that the cells fluoresced red, indicating the presence of propidium iodide; however, the membranes were compromised. These results indicated that the artificial concentration of *S. marcescens* WW4 in BM medium induced major cell death (but not lysis); however, the presence of *P. aeruginosa* WW5 remarkably increased the ability of concentrated *S. marcescens* WW4 to survive under phosphate limitation.

**Table 2:** The viability of concentrated *Serratia marcescens* WW4 when co-cultured in the presence and absence of *Pseudomonas aeruginosa* WW5 in BM medium

	Viability (mean ± standard error) of WW4 cells (%)		
	
Initial OD_600_ of WW4	Without WW5	With WW5	*P*
0.1	52.13 ± 0.34	54.46 ± 0.15	ns
0.2	10.64 ± 0.03	23.01 ± 0.02	ns
0.3	0.08 ± 0.00	11.82 ± 0.02	**
0.4	0.01 ± 0.00	9.95 ± 0.05	*
0.5	0.00 ± 0.00	0.99 ± 0.01	*
0.6	0.00 ± 0.00	0.35 ± 0.00	*

Significant differences (two-tail *t*-test, *P < 0.05 and **P < 0.001) are indicated for the viability of concentrated *Serratia marcescens* WW4 growing in the presence and absence of *Pseudomonas aeruginosa* WW5 in BM medium after 24 h.

ns, not significant.

In this experiment, concentrated *S. marcescens* WW4 cells may be abnormal under phosphate limitation. *Serratia marcescens* WW4 might physiologically respond to phosphate levels, with a certain level being sufficient for the survival of the population. Otherwise, *S. marcescens* WW4 might turn on a death pathway to control population biomass. This phenomenon might follow a process similar to the quorum sensing system that is involved in monitoring the population density of bacteria and up-regulating the production of virulence factors (Van Houdt *et al*., 2007). This phosphate-limited inhibition might also be associated with the growth-phase-dependent manner and SOS regulation that are involved in the bacterial inhibition of *S. marcescens* MG1 by producing nuclease bacteriocin (Guynn *et al*., 1998). As *P. aeruginosa* WW5 coexists with *S. marcescens* WW4 under phosphate limitation, *S. marcescens* WW4 might inhibit the growth of *P. aeruginosa* WW5 to delay or reduce massive programmed death through quorum sensing signalling or SOS responses. Another possibility is that *P. aeruginosa* WW5 might be more sensitive to the mechanism of cell density control by *S. marcescens* WW4. The substances from *S. marcescens* WW4 might accidentally cause *P. aeruginosa* WW5 to die in place of *S. marcescens* WW4 under phosphate limitation.

### A bacteriocin-attenuated mutant of *S. marcescens* WW4

**Fig. 3:**
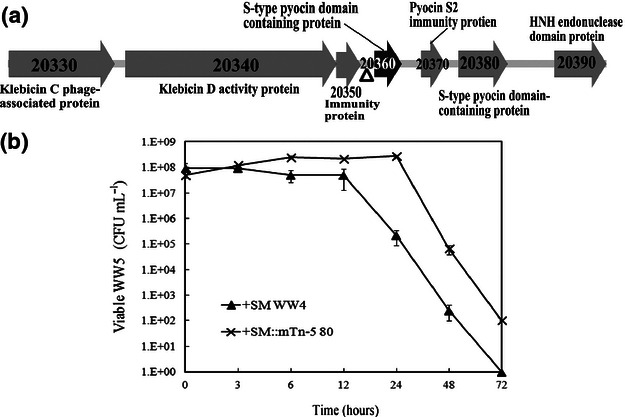
The transposon insertion mutant of *Serratia marcescens* WW4. The diagram (a) shows the inserted transposon-mediated locus on the chromosome of the mini Tn-5 mutant (SM::mTn-5 80) that was associated with phosphate-limited inhibition. The inserted site (▵) was located upstream of a bacteriocin (CDS SMWW4_v1c20360), which was similar to an S-type pyocin with endonuclease activity. The genomic sequences were sequenced by Illumina sequencing and annotated by blastp in GenBank. The viability of *Pseudomonas aeruginosa* WW5 co-cultured with *S. marcescens* WW4 (SM WW4) or SM::mTn-5 80 in BM medium is presented in (b). The data show the means of viable *P. aeruginosa* WW5 cell numbers (CFU mL^−1^). The bars indicate the standard error.

To understand the mechanism of phosphate limitation-induced inhibition of *P. aeruginosa* WW5 by *S. marcescens* WW4, a library of random insertional mutants was created in *S. marcescens* WW4 using the mini-Tn5 transposon method (De Lorenzo *et al*., 1990; Winson *et al*., 1998). We analysed the inhibitory effect of these mutants against *P. aeruginosa* WW5, as described in Materials and methods. The SM::mTn-5 80 mutant was screened based on a lack of inhibitive ability against *P. aeruginosa* WW5 in the BM medium co-culture after 24 h. The analysis of genomic DNA in the SM::mTn-5 80 mutant at the insertion site indicated that the mini-Tn5 transposon was located upstream of the predicted protein-coding sequence (CDS) SMWW4_v1c20360 (Fig. 3a). The insertion site (△) was 14 bp upstream of the predicted transcriptional start site and just in front of the ribosome binding site (rbs). The promoter location of this gene was predicted 136–186 bp in front of the start codon by the Neural network promoter prediction (Reese, 2001). Therefore, this promoter in the SM::mTn-5 80 mutant was separated from the rbs and start codon by the mini-Tn5 insertion. The protein blastp result in the NCBI GenBank showed that the corresponding amino acid sequence was similar to an S-type pyocin bacteriocin with endonuclease activity. The closest predicted proteins were the pyocin_S region in a putative klebicin D activity protein of *Erwinia tasmaniensis* Et1/99 (GenBank accession number CAO94994, 65% identity at protein level) and an S-type pyocin domain–containing protein in *Serratia odorifera* 4Rx13 (GenBank accession number EFA14144, 54% protein sequence identity). According to the blast result, we assumed that the SM::mTn-5 80 mutant is a bacteriocin-deficient mutant.

Furthermore, the neighbouring regions of this putative bacteriocin contain six other genes related to bacteriocin (Fig. 3a). For example, CDS SMWW4_v1c20330 is similar to a klebicin C phage-associated protein of *E. tasmaniensis* Et1/99 (CAO94993). CDS SMWW4_v1c20340 is also similar to the pyocin_S and colicin-DNase regions in a klebicin D protein of *E. tasmaniensis* Et1/99 (CAO94994). CDS SMWW4_v1c20350 is similar to an immunity protein of *Pectobacterium carotovorum* subsp. *carotovorum* (ADH95193). CDS SMWW4_v1c20370 is highly similar to a pyocin S2 immunity protein of *Yersinia pseudotuberculosis* IP 32953 (YP_068698). CDS SMWW4_v1c20380 is similar to an S-type pyocin domain-containing protein of *S. odorifera* 4Rx13 (ZP_06193368). Finally, CDS SMWW4_v1c20390 is highly similar to an HNH endonuclease domain protein of *S. odorifera* 4Rx13 (ZP_06193370).

Distinct from general pore-forming bacteriocins, S-type pyocins (S1, S2, S3, AP41) have been identified to exhibit endonuclease activity, which causes cell death by degrading DNA inside sensitive cells, without the lysis of target cells (Michel-Briand & Baysse, 2002). This phenomenon might explain why *S. marcescens* WW4 only inhibited the growth of *P. aeruginosa* WW5, but did not lyse the cells, as indicated by propidium iodide nucleic acid staining. An S-type pyocin generally contains three functional domains, including the N-terminal receptor binding domain, the outer-membrane translocation domain and the C-terminal domain carrying DNase activity (Sano *et al*., 1993). Following the C-terminal end of S-type pyocins, the small protein homologous to colicin E2 is identified as an immunity protein that protects host bacteria from DNA breakdown (Michel-Briand & Baysse, 2002). As it is often observed in pyocin and colicin systems (Michel-Briand & Baysse, 2002; Cascales *et al*., 2007), the small immunity protein gene (CDS SMWW4_v1c20370) was located immediately adjacent to the putative S-type pyocin (CDS SMWW4_v1c20360). This immunity protein may protect the host by binding to the nuclease bacteriocin prior to its release and thus preventing toxic effects. In addition, four other genes related to the bacteriocin activity were located near to the putative S-pyocin in the same chromosomal region and may be involved in the inhibitive ability of *S. marcescens* WW4.

To examine the bacteriocin mutant, we also assayed the inhibitive ability of SM::mTn-5 80 in BM medium for 3 days. When co-culturing *P. aeruginosa* WW5 with the SM::mTn-5 80 mutant in BM medium, the viability of *P. aeruginosa* WW5 remained above 10^8^ CFU mL^−1^ for 24 h (Fig. 3b). At 48 h of incubation, the viability of *P. aeruginosa* WW5 cells was much higher when co-cultured with the SM::mTn-5 80 mutant compared with *S. marcescens* WW4. This finding indicates that the inhibitory effect of this mutant was lower than the wild-type *S. marcescens* WW4. Because the inserted site was located between the predicted promoter region and the rbs/start codon region of CDS SMWW4_v1c20360, but not within the coding region, it may be hypothesised that the transposon insertion in the SM::mTn-5 80 mutant only partially disrupts the function of this bacteriocin gene. Ideally, a complementation experiment would provide unambiguous evidence for the function of this bacteriocin gene. However, despite several attempts, we have not been able to clone this gene into the *E. coli* system, possibly due to the lethality of this bacteriocin gene. Nonetheless, the available evidence (e.g. mutant phenotype, bioinformatic analysis of this gene, and its neighbouring regions on the chromosome) strongly support our hypothesis that this endonuclease bacteriocin is involved in the observed intergeneric inhibition by *S. marcescens* WW4 under phosphate limitation.

Previous studies have frequently reported that *Serratia* spp. inhibit the growth of Gram-negative bacteria when using broad host-spectrum antibiotics, such as carbapenem and the red pigment prodigiosin (Wei & Lai, 2006). Theoretically, *S. marcescens* WW4 should produce such general antibiotics to inhibit the growth of *P. aeruginosa* WW5 when competing for a space and nutrients in an ecosystem. However, our results indicate that *S. marcescens* WW4 induces a species-specific bacteriocin to compete with *P. aeruginosa* WW5 under phosphate limitation. Bacteriocins often act on closely related bacteria; however, *S. marcescens* WW4 and *P. aeruginosa* WW5 are classified in different taxonomical orders. We assume that the nuclease bacteriocin of *S. marcescens* WW4 is very similar to a pyocin from *P. aeruginosa*-related bacteria. The *S. marcescens* WW4 bacteriocin induced by phosphate limitation might completely inhibit the growth of *P. aeruginosa* WW5 when *S. marcescens* WW4 receives a signal from a quorum sensing or SOS system. When phosphate is insufficient, *S. marcescens* WW4 might also induce genes required for phosphate uptake, such as the histidine kinase SphS, the alkaline phosphatase phoA and the extracellular nuclease nucH in the cyanobacterium *Synechocystis* sp. PCC 6803 (Michel-Briand & Baysse, 2002). Hence, to obtain phosphate from the external environment, the function of the endonuclease bacteriocin of *S. marcescens* WW4 might be similar to that of the extracellular nuclease nucH.

## Conclusion

Our results demonstrated that a putative endonuclease bacteriocin in *S. marcescens* WW4 was involved in an inhibitory effect across different bacterial genera under phosphate-limited conditions. Our findings led us to assume that when *S. marcescens* WW4 and *P. aeruginosa* WW5 inhabit industrially processed water, in which nutrients and phosphate are limited, the two bacteria are forced to compete for space and nutrients to survive. Under phosphate limitation, *S. marcescens* WW4 inhibits and out-competes *P. aeruginosa* WW5 by producing an endonuclease bacteriocin, which usually acts on interspecific interactions. This intergeneric inhibition prolongs the viability and biofilm structure of *S. marcescens* WW4 in the bacterial ecosystem.

## Supporting Information

**Fig. S1.** The viability of *S. marcescens* WW4 and *P. aeruginosa* WW5 in pure or co-culture chambers.
