# Supplementary figures and images for: Phosphate limitation induces the intergeneric inhibition of Pseudomonas aeruginosa by Serratia marcescens isolated from paper machines
Source: FEMS Microbiol Ecol. 2013 Mar 11;84(3):577–87. doi: 10.1111/1574-6941.12086 (PMC3717176; doi:10.1111/1574-6941.12086)

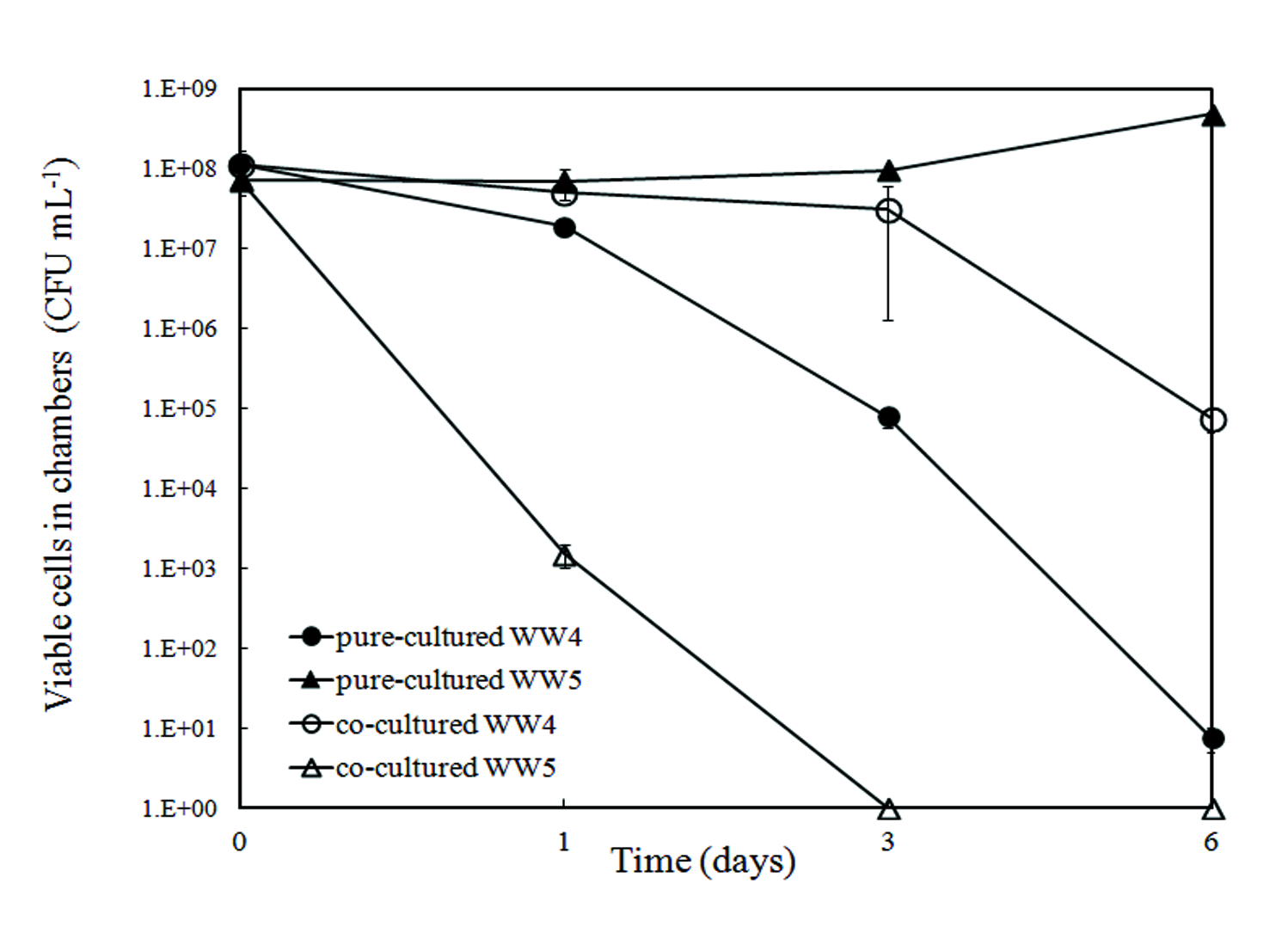

Supplement: Supplementary file 1 [file fem0084-0577-SD1.tif]
